# Rapid Detection and Identification of Overdose Drugs in Saliva by Surface-Enhanced Raman Scattering Using Fused Gold Colloids

**DOI:** 10.3390/pharmaceutics3030425

**Published:** 2011-07-13

**Authors:** Stuart Farquharson, Chetan Shende, Atanu Sengupta, Hermes Huang, Frank Inscore

**Affiliations:** Real-Time Analyzers, Inc., Middletown, CT 06457, USA; E-Mails: chetan@rta.biz (C.S.); atanu@rta.biz (A.S.); hermes@rta.biz (H.H.); inscore@rta.biz (F.I.)

**Keywords:** drug analysis, illicit drugs, drug overdose, SERS, Raman spectroscopy, emergency room

## Abstract

The number of drug-related emergency room visits in the United States doubled from 2004 to 2009 to 4.6 million. Consequently there is a critical need to rapidly identify the offending drug(s), so that the appropriate medical care can be administered. In an effort to meet this need we have been investigating the ability of surface-enhanced Raman spectroscopy (SERS) to detect and identify numerous drugs in saliva at ng/mL concentrations within 10 minutes. Identification is provided by matching measured spectra to a SERS library comprised of over 150 different drugs, each of which possess a unique spectrum. Trace detection is provided by fused gold colloids trapped within a porous glass matrix that generate SERS. Speed is provided by a syringe-driven sample system that uses a solid-phase extraction capillary combined with a SERS-active capillary in series. Spectral collection is provided by a portable Raman analyzer. Here we describe successful measurement of representative illicit, prescribed, and over-the-counter drugs by SERS, and 50 ng/mL cocaine in saliva as part of a focused study.

## 1. Introduction

In 2009 the number of drug-related emergency room (ER) visits in the United States reached 4.6 million, nearly double the 2.4 million visits in 2004 [1]. Of these visits, 2.1 million were attributed to illicit drugs and 2.3 million to pharmaceutical drugs. Access to prescription drugs over the internet has certainly added to the increase in the latter. Cocaine and heroin represent the largest portion of illicit drugs at 43 and 22%, respectively, followed by methamphetamine, PCP, MDMA (known as ecstasy), and LSD. Pharmaceutical drugs include prescription and over-the-counter drugs (OTC), leading prescription drugs being oxycodone, hydrocodone and diazepam at 14.1, 8.4, 2.4%, respectively, and leading OTC drugs being acetaminophen and ibuprofen at 4.8, 2.7%, respectively. Overdose represents a significant challenge to ER personnel, since it can result in a variety of symptoms [2], confusing diagnosis. For example, cocaine may cause myocardial infarction, hypothermia, seizures, hallucinations, and/or arrhythmias [3]. Acetaminophen overdose symptoms include abdominal pain, convulsions and sweating [4]. Physical assessment of these patients is insufficient, especially in the case of acetaminophen, as most patients are over 65 and have existing health issues. Currently, a two step approach is used to determine drug abuse and overdose: screening and verification [3,5]. Screening devices, such as immunoassay kits, provide drug identification, but they are subject to a number of interferences [6,7]. This leads to a high incidence of false positives. Therefore, sophisticated instruments, such as gas chromatographs coupled with mass spectrometers, are used to verify drug identification, as well as provide quantification [8]. Unfortunately, these analyses, performed in the clinical laboratory, usually take in excess of an hour to perform [9], delaying patient diagnosis and selection of appropriate medical care.

Consequently, there is a need for a device capable of correctly identifying overdose drugs in ER patients at relevant concentrations, *i.e.*, above a threshold, without false positives or negatives, in just a few minutes. Ideally, the device would be portable, easy to use, and relatively non-invasive so that patients can be tested as they enter the ER, or in an ambulance prior to arrival. The last requirement can be best met using saliva as the sample medium [10]. This is a reasonable approach since drugs are represented in saliva at concentrations similar to blood plasma (e.g., cocaine at 0.6–0.8 mcg/mL [11-13] acetaminophen at 10–50 mcg/mL [14]), saliva is 99.5% water making it easy to chemically analyze [15], and simple saliva collectors are available [16].

In an effort to develop such a device, and take advantage of saliva as a sample source, we have been investigating the potential of surface-enhanced Raman spectroscopy (SERS) to both identify and quantify drugs and their metabolites in this body fluid [17,18]. This approach is based on the extreme sensitivity of SERS demonstrated by the detection of single molecules [19,20], the ability to measure very small samples (0.1 mL), and the ability to identify molecular structures of drugs through the rich vibrational information provided by Raman spectroscopy [21].

Towards this goal, we have developed and patented a method to immobilize fused silver and gold colloids in a porous glass matrix in glass capillaries to produce a SERS-active sampling device [22-24]. In contrast to most SERS substrates, the sample does not have to be dried to achieve enhancement [25], but can be drawn into the capillary and measured immediately. Furthermore, the capillary format allows the incorporation of chromatographic materials to aid in the separation of the drugs from saliva. Here we describe the use of a solid-phase extraction (SPE) capillary in combination with a SERS-active capillary to detect and identify cocaine, PCP, diazepam, and acetaminophen in saliva using a portable Raman analyzer suitable for emergency room diagnosis of overdose patients.

## 2. Experimental Section

**Figure 1.**
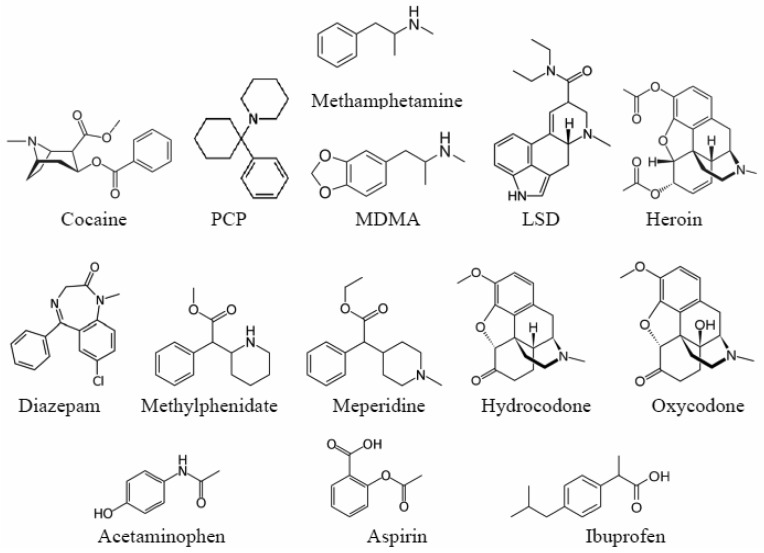
Chemical structures for the drugs presented in this study.

All drugs were obtained from Cerilliant (Austin, TX) as 1 mg/mL in methanol or acetonitrile forensic samples (Figure 1, Table 1). The chemicals and solvents used to prepare the sol-gels were obtained at their purest commercially available grade from Sigma-Aldrich (Milwaukee, WI) and used as received. The SPE material was obtained from United Chemical Technology (Bristol, PA). The fused gold colloid sol-gels were prepared according to previous published procedures [26,27], by mixing a gold chloride salt (HAuCl_4_•3H_2_O) with tetramethyl orthosilicate in methanol. The SERS capillaries were prepared by drawing 20 μL of the gold colloid-doped sol-gels into 10 cm long, 1-mm diameter glass capillaries to produce ∼1 cm plugs. After sol-gel formation, the incorporated ions were reduced with dilute sodium borohydride forming fused colloids. This was followed by a water wash to remove residual reducing agent.

Solid-phase extraction packed capillaries (also 1-mm diameter glass) were prepared by first drawing ∼5 mcL of methyltrimethoxysilane (MTMS) into one end of a glass capillary by syringe to form a porous sol-gel frit as a support for the SPE material. Secondly, ∼100 mcL of a 20 mg/mL SPE/ethanol slurry was drawn into the capillary to form an ∼5 cm plug. And thirdly, a second 5 mcL of MTMS was drawn into the capillary to secure the SPE material. The SPE capillary was preconditioned by sequentially flowing 1 mL each of methanol, water and 10 mM acetic acid. Each experiment was performed by drawing the test sample through the SPE capillary, then washing the capillary to remove any interferents, and just prior to the final elution step, the SERS capillary was attached to the SPE capillary so that the extracted drugs could be eluted directly into the SERS capillary.

Saliva used in preparing artificial samples was collected using oral swabs (Medimpex United Inc, Bensalem, PA) from consenting employees at Real-Time Analyzers, Inc. (RTA, Middletown, CT). The foam head attached to a syringe plunger was placed into the person's mouth for approximately one minute to let sufficient saliva collect in the foam. The swab was then placed into the plastic syringe barrel, and pressed to expel the saliva into a vial. Approximately 1 mL of saliva was collected in 1 minute by this process.

The drug-doped saliva samples were prepared in plastic centrifuge tubes by adding a small amount of aqueous drug at the required concentration into the saliva and uniformly mixed by gently vortexing the sample for 10 seconds. For example, 10 mcL of a 5 mcg/mL aqueous cocaine was added to 0.990 mL of saliva to yield a 1 mL 50 ng/mL cocaine-doped saliva sample. The samples were allowed to equilibrate for 0.5 hours at room temperature. Samples were measured within 1 hour of saliva collection. Approximate concentrations were verified by gas chromatography (Shimadzu, model 17A).

**Figure 2.**
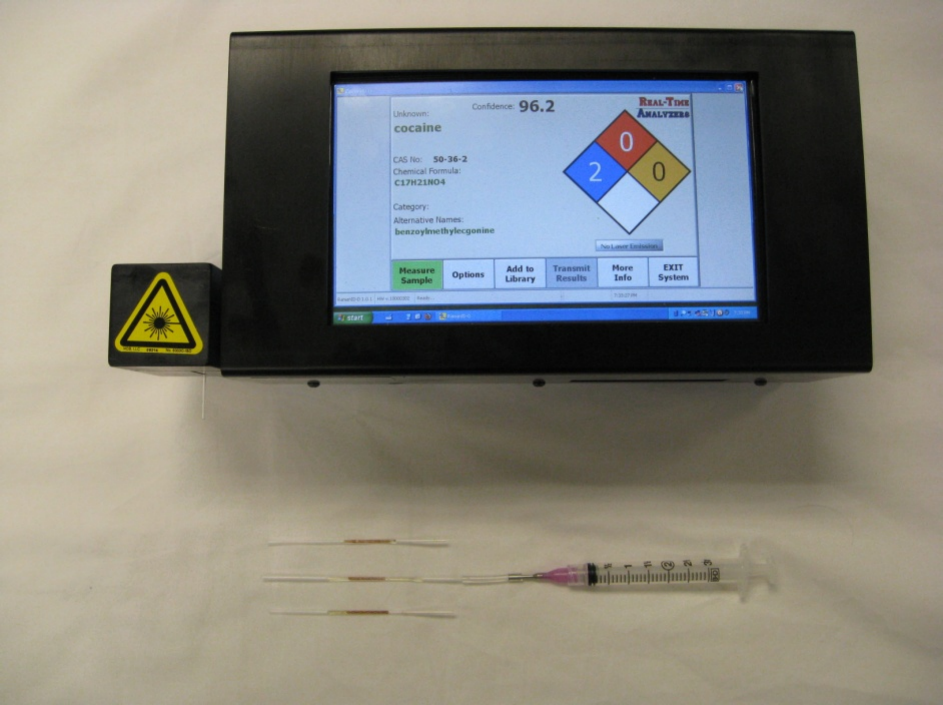
Photographs of surface-enhanced Raman spectroscopy (SERS)-ID and SERS-active capillaries.

A searchable surface-enhanced Raman spectra library was prepared by measuring all of the drugs using a Fourier transform Raman spectrometer (RTA, model RamanPro) that provided 100 to 3350 cm^−1^ spectral coverage with constant 8 cm^−1^ resolution. The SERS-active capillaries were fixed horizontally to an XY positioning stage (Conix Research, Springfield, OR) mounted above a fiber optic probe. The probe delivered 75 mW of 785 nm laser excitation to the capillary and collected the 180° scattered radiation. A pure cocaine sample was prepared by drying a forensic sample on a glass slide. The Raman spectrum of this residue was measured using 300 mW of 785 nm laser power and a 5 minute acquisition time. Further instrument details have been published [21]. To demonstrate ER measurement capability, the drug-doped saliva samples were also measured using a 5 lb, battery operated, hand-held Raman analyzer (RTA, model SERS-ID, Figure 2). The SERS capillaries were fixed horizontally in the sample compartment of the portable analyzer, which delivered 45 mW of 785 nm excitation laser to the capillary and collected the 180° scattered radiation. In the case of all the spectra presented here, three capillaries were measured and the average spectrum reported.

## 3. Results and Discussion

**Figure 3.**
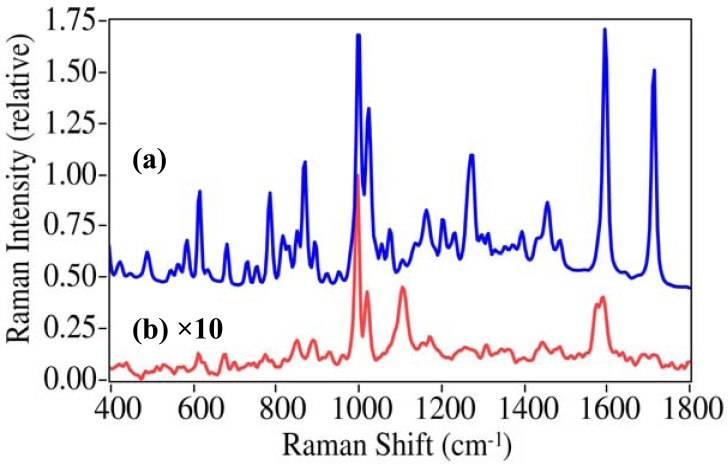
Comparison of **(a)** Raman spectroscopy (RS) of pure cocaine on a glass slide to **(b)** SERS for 100 ng/mL (100 ppb) cocaine in water (as cocaine•HCl) using a fused gold colloid-doped sol-gel in a 1 mm capillary. Conditions: **(a)** 300 mW of 785 nm, 5 minute acquisition, **(b)** 75 mW of 785 nm excitation, 1 minute acquisition; both at 8 cm^−1^ resolution. The SERS spectral intensity has been multiplied by 10 so that features are evident, and offset for clarity.

One hundred and fifty drugs; illicit, prescription, over-the-counter, and in many cases their primary metabolites, were measured by SERS using fused gold colloids immobilized within a sol-gel matrix contained in glass capillaries. The gold colloids enhanced the Raman scattering for virtually all of the drugs tested, with some barbiturates as the primary exception. In many cases the normal Raman spectra were also measured to verify spectral integrity as well as to estimate surface enhancement factors (EF). This is shown for cocaine, the primary drug of concern (Figure 3) [1]. The Raman spectrum of cocaine is dominated by peaks at 872, 999, 1026, 1273, 1597, and 1716 cm^−1^, which have been assigned to a tropine ring stretch, the symmetric and asymmetric phenyl ring breathing modes, the C-phenyl stretch, the trigonal phenyl ring breathing mode, and the ester carbonyl stretch [28,29]. There are clear changes in relative intensity and frequency for these modes in the SER spectra due to the extent with which each vibrational mode interacts with the gold surface and the plasmon field [17]. Notably, the symmetric mode at 999 cm^−1^ is enhanced the most, the asymmetric mode shifts to 1018 cm^−1^, a new unassigned peak appears at 1107 cm^−1^, the trigonal mode looses relative intensity as its splits into a 1578 and 1593 cm^−1^ doublet, and the carbonyl stretch is virtually absent. An enhancement factor of 3.1 × 10^6^ was determined based on the relative concentrations, intensities of the 999 cm^−1^ peak, and laser powers for the two spectra (EF = [1 g/mL/100 ng/mL] × [0.1 I/1.3 I] × [300 mW/75 mW]). EFs were typically between 10^5^ and 5 × 10^6^ for the drugs presented in this paper.

**Figure 4.**
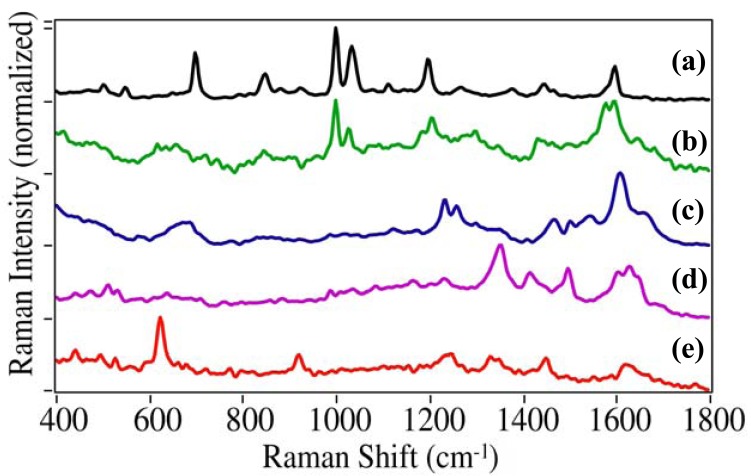
SERS of illicit drugs: **(a)** 1-(1-phenylcyclohexyl) piperidine (PCP), **(b)** methamphetamine, **(c)** 3,4-methylenedioxymethamphetamine (MDMA), **(d)** lysergic acid diethylamide (LSD), and **(e)** heroin. Conditions as in Figure 3(b), except at 0.1 mg/mL. Ordered to show spectral similarity. Intensities normalized and offset for clarity.

In addition to cocaine, this study focused on the illicit, prescription and OTC drugs that most often result in ER visits [1]. The additional illicit drugs included PCP (1-(1-phenylcyclohexyl) piperidine), dl-methamphetamine, MDMA (3,4-methylenedioxymethamphetamine), LSD (lysergic acid diethylamide), and heroin (diacetylmorphine). Each drug produces a unique SERS spectrum that can be used for identification (Figure 4). While the spectra for cocaine, PCP, and methamphetamine are dominated by the phenyl stretching modes at 999, ∼1030 and ∼1595 cm^−1^, they each have unique peaks: 1107 cm^−1^ for cocaine, 702 and 849 cm^−1^ due to the cyclohexane and piperidine ring modes for PCP, respectively, and 1578 cm^−1^ phenyl mode for methamphetamine [30,31]. The phenyl modes are absent as expected for MDMA, LSD, and heroine [32]. MDMA has a unique doublet at 1233 and 1258 cm^−1^ due to the dioxole ring and a C-N stretching mode, LSD at 1350 cm^−1^ also due to a ring or C-N stretching mode, and heroin at 624 and 921 cm^−1^ due to ring modes.

**Figure 5.**
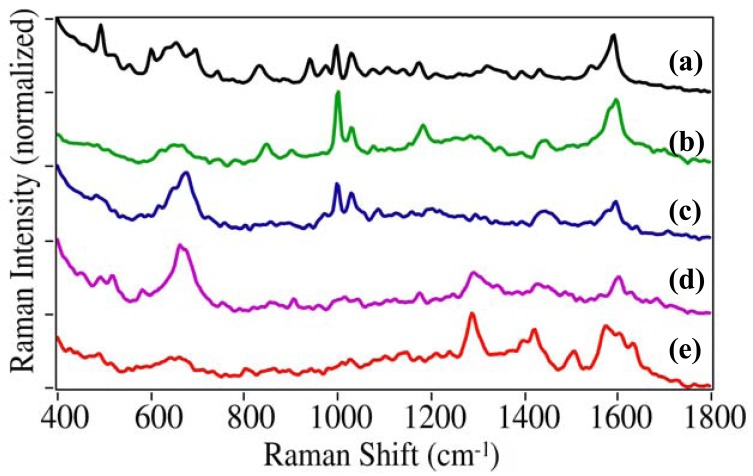
SERS of prescription drugs: **(a)** diazepam (Valium®), **(b)** methylphenidate (Ritalin®), **(c)** meperidine (Demerol®), **(d)** hydrocodone (Vicodin®), and **(e)** oxycodone (Oxycotin®). Conditions as in Figure 4. Ordered to show spectral similarity. Intensities normalized and offset for clarity.

The SERS spectra were measured for prescription drugs with the following active ingredient (Figure 5): diazepam (Valium®), methylphenidate (Ritalin®), meperidine (Demerol®), hydrocodone (Vicodin®), and oxycodone (Oxycotin®). Identifying unique peaks for these drugs is more difficult than the illicit drugs due to their structural similarity. Once again the phenyl modes are present at ∼1000, 1030, and 1595 cm^−1^ for diazepam, methylphenidate, and meperidine, but as expected, not for hydrocodone and oxycodone. A number of unique peaks appear between 600 and 700 cm^−1^, as well as at 833 and 941 cm^−1^ for diazepam, all attributed to aromatic ring deformation modes, except the 833 cm^−1^ peak, which is attributed to the diazapine ring [33]. Although methylphenidate and meperidine have nearly identical structures, the piperidine ring of the former appears to interact with the gold through the amine resulting in two peaks at 849 and 903 cm^−1^. The spectra of hydrocodone and oxycodone are very similar with C=C ring modes at 1293 and ∼1605 cm^−1^. The former has a unique doublet at 664 and 675 cm^−1^, which is attributed to ring modes as was done for heroin, while the latter has a unique peak at 1505 cm^−1^ that has been attributed to a scissoring mode of the methyl group [34].

**Figure 6.**
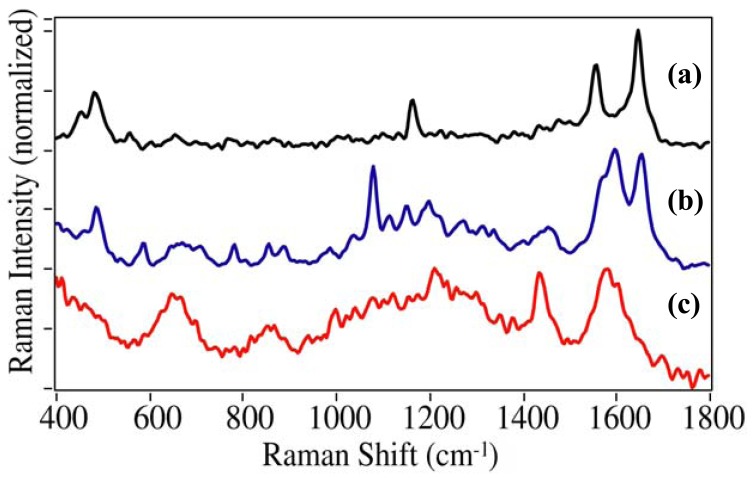
SERS of OTC drugs: **(a)** acetaminophen, **(b)** aspirin, and **(c)** ibuprofen. Conditions as in Figure 4. Intensities normalized and offset for clarity.

The OTC drugs included acetaminophen, aspirin (acetylsalicylic acid), and ibuprofen. While acetaminophen and ibuprofen have been implicated in numerous overdose cases (often unintentionally), aspirin has been included in this study as it is often taken with many of the other drugs. Again, each drug has unique peaks suitable for identification. For acetaminophen this includes the 1165, 1555, and 1647 cm^−1^ phenol, C=C ring, and amide stretches, respectively (Figure 6). For aspirin this includes numerous unassigned peaks between 500 and 900 cm^−1^, the 1080 cm^−1^ ortho-substituted ring mode, and the 1655 cm^−1^ ester mode. For ibuprofen a COOH bending mode appears at 1439 cm^−1^. Aspirin and ibuprofen also have COOH stretching modes at 1597 and 1582 cm^−1^, respectively.

**Figure 7.**
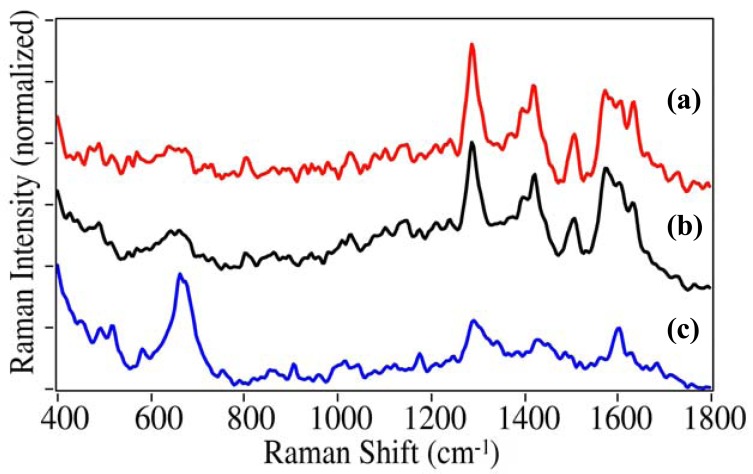
SERS of **(a)** oxycodone as an unknown, **(b)** oxycodone in the spectral library, and **(c)** hydrocodone as the second best match in the spectral library. The Hit Quality Index (HQI) scores were **(b)** 0.073 and **(c)** 0.734, indicating **(a)** as the best match. Conditions as in Figure 4.

While the traditional method of identifying unique peaks can be used to identify drugs as described above, a more reliable method is to compare the entire spectrum to a library of previously recorded spectra for identification. This not only eliminates the tedium of identifying peaks, but also allows the identification process to be automated. Four algorithms, Absolute Value, Least Squares, Euclidean Distance, and Correlation, were examined for this purpose [35]. In essence the first two algorithms subtract the measured spectrum from each library spectrum, such that an exact match would equal zero, while the second two algorithms divide the measured spectrum by each library spectrum, such that an exact match would equal one. However, the latter two algorithms subtract the results from one to conform to the first two algorithms. In all cases, the results are reported as the Hit Quality Index (HQI) where a perfect match and complete mismatch (no peaks in common) would result in HQIs of 0 and 1, respectively. Here the spectra were pretreated by restricting the spectral region from 400 to 1800 cm^−1^ and setting the baseline intensity to zero and the most intense peak to one. The Correlation algorithm proved best, and the results are shown for a measurement of oxycodone as an unknown compared to the 152 drug spectral library (Table 1, Figure 7). Even though this drug has the least unique SERS spectrum presented here, it is easily identified as the best match with an HQI score of 0.073, and hydrocodone, the next best match, has an HQI score of 0.734, clearly a mismatch (Table 2). The success of this method is very similar to a previous study of 309 pharmaceuticals by Raman spectroscopy [36].

Previous measurements of saliva samples artificially doped with cocaine demonstrated a measured limit of 250 mcg/mL [17]. This is not surprising since salivary mucins (a class of high molecular weight glycosylated proteins) can physically and in some cases chemically bind the drugs so that they are unavailable for enhancement at the metal colloid surfaces [37,38]. Furthermore, the highly viscous nature of these mucins can clog the sol-gel pores, as well as block the metal surface. In an effort to make the drugs available for SERS, a simple method suitable to the ER was developed to break-up the mucins and release the drugs from proteins using acetic acid, and to separate the drugs for analysis using solid-phase extraction. As part of this method development, SPE capillary columns were prepared using 1-mm glass capillaries to match, and be used in-line with, the SERS-active capillaries. The SPE material chosen consisted of silica particles functionalized with octyl groups and benzyl sulfonic acid groups, as this material has been successfully used to separate numerous drugs, including cocaine from biological fluids [39]. The final method consisted of the following steps. 1) A 1 mL saliva sample (500 mcL of saliva plus 500 mcL of 10 mM acetic acid) was drawn through a 1 micron filter into a SPE capillary column, depositing the drug and passing some of the saliva components. 2) 1 mL each of 10 mM acetic acid, methanol and water were drawn through the SPE capillary column removing any residual saliva components. 3) A 20 mcL 2% ammonium hydroxide in acetonitrile solution was drawn through the SPE capillary column into a SERS capillary, extracting the drug from the SPE, and depositing it on the fused gold colloid-doped sol-gel. 4) The SERS capillary was placed in the sample compartment of the portable Raman analyzer and measured. The entire process took less than 10 minutes for each sample.

**Figure 8.**
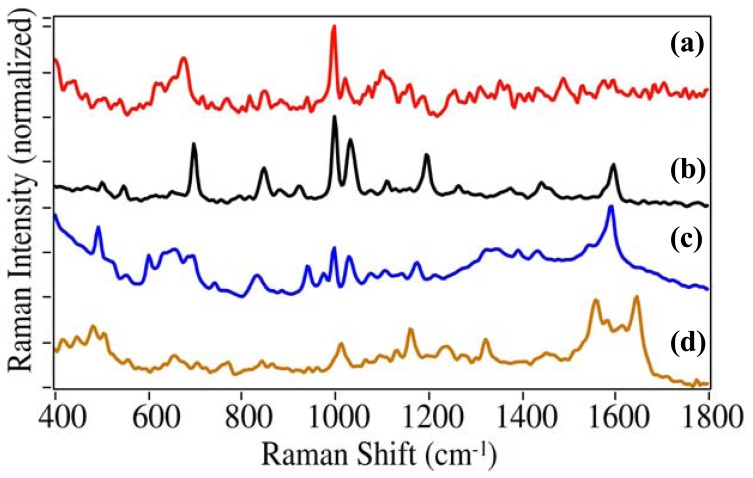
SERS of **(a)** 50 ng/mL cocaine, **(b)** 1 mcg/mL PCP, **(c)** 1 mcg/mL diazepam, and **(d)** 10 mcg/mL acetaminophen extracted from saliva. Conditions as in Figure 4, except at indicated concentrations.

Using this procedure, a series of saliva samples doped with cocaine, PCP, diazepam, and acetaminophen were prepared beginning at 1 mg/mL, then diluted by factors of 10 to 1 mcg/mL and measured. In the case of cocaine, the dilution was continued to 10 ng/mL. While cocaine, PCP and diazepam produced consistent SERS at 1 mcg/mL and above, acetaminophen was sporadic at this concentration, but consistent at 10 mcg/mL and above (Figure 8).

**Figure 9.**
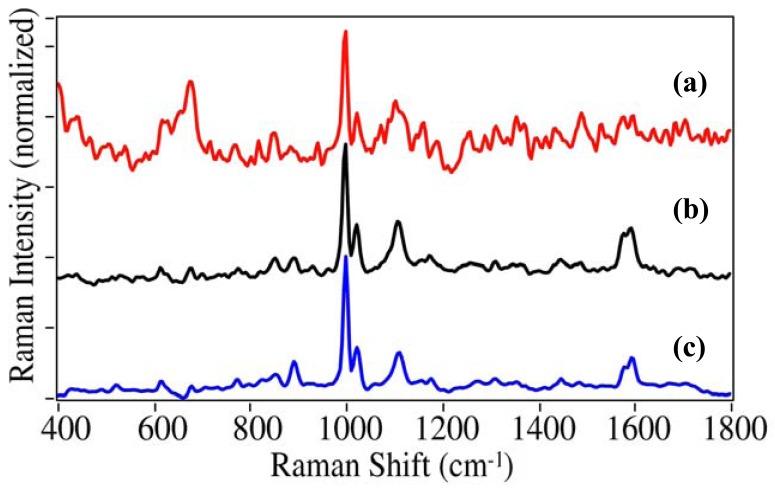
SERS of **(a)** 50 ng/mL cocaine in saliva as an unknown, **(b)** 0.1 mg/mL cocaine in the spectral library, and **(c)** ethylbenzoylecgonine as the second best match in the spectral library. The HQI scores were **(b)** 0.287 and **(c)** 0.348. Conditions as in Figure 4, except at indicated concentrations.

In the case of cocaine, for which the above method was optimized, it was consistently detected at 50 ng/mL and higher, and sporadically at 10 ng/mL. These sensitivities demonstrated for cocaine and acetaminophen are sufficient to detect typical overdose saliva concentrations of ∼0.6–0.8 and 10–50 mcg/mL for these drugs, respectively [13,14]. It should be noted that the analyzer does not have to quantify the drugs, but it does need to have sufficient sensitivity to detect and subsequently identify them. The ability of the analyzer and the developed method to perform this task is shown in Figure 9 for cocaine at 50 ng/mL, the reproducible limit of detection. The measured sample was compared to the 152 drug spectral library, and as can be seen, the best match is the library spectrum of cocaine. However, the HQI score is relatively high due to the lower signal-to-noise ratio, and the scores for the next best matches were close at 0.348 and 0.349 for ethylbenzoylecgonine and benzoylecgonine, both metabolites of cocaine with near identical structures (Table 2). The former is formed in the presence of alcohol in the body by replacing the methyl group of the methyl ester with an ethyl group. The latter is formed as part of the metabolic breakdown of cocaine by replacing the methyl group of the methyl ester with a hydrogen. As mentioned above for cocaine, the ester modes are very weak in the SERS spectra, and consequently, the spectra are very similar. Considerably better results were obtained for PCP, diazepam, and acetaminophen, albeit at higher concentrations (Table 2). In all cases the HQI score of the second best match was considerably higher (worse) than the first.

## 4. Conclusions

Surface-enhanced Raman spectra of 152 drugs and metabolites were successfully collected using fused gold colloids immobilized in a porous glass matrix contained in glass capillaries. Spectra are shown for a series of illicit, prescription, and OTC drugs. A method was successfully developed to measure such drugs in saliva by combining a solid-phase extraction capillary to separate the drugs from saliva with the SERS-active capillary. The method allowed the detection and identification of 50 ng/mL cocaine, 1 mcg/mL PCP, 1 mcg/mL diazepam, and 10 mcg/mL acetaminophen in saliva using a 152 spectral library and a search and match software program. The entire analysis, from sample collection to positive identification was performed in less than 10 minutes. The cocaine measurement represents a vast improvement compared to a previously reported SERS measurement of cocaine in saliva; the measurement was 5000-times better [17]. Future work will develop the capillaries into a lab-on-a-chip as part of a sample kit to be used by ambulance and emergency room personnel in conjunction with a hand-held Raman analyzer.

## Figures and Tables

**Table 1.** Drugs measured to build a spectral library. Drugs presented in this study are in bold type. Metabolites are italicized.

*11-nor-Ä^9^-THC-9-COOH*	**Cocaine HCl**	Lactose	Penacillamine
*11-OH-Ä^9^-THC*	Codeine	l-amphetamine	Phenacetin
*6-acetylcodeine*	d-amphetamine (Dexedrine)	l-ephedrine HCl	Phenethylamine
*6-acetylmorphine*	Ä^9^-THC	Levamisole (Ergamisol)	Phenobarbital
**Acetaminophen**	Dextromethorphan	Lidocaine (lignocaine)	Phentermine
Acetylcholine chloride	**Diazepam (Valium)**	Lorazepam (Ativan)	Phenylephrine
**Acetylsalicylic acid (aspirin)**	Dimenhydrinate (Dramamine)	**LSD**	Phenylpropanolamine HCl
Alfentanil HCl	Diphenhydramine	Mannitol	*PMA HCl*
Alprazolam (Xanax)	dl-amphetamine	MDA	*PMMA HCl*
Amifostin	dl-ethylamphetamine	MDEA	Pregabalin (Lyrica)
Aminorex	**dl-methamphetamine**	**MDMA**	Prilocaine
Amitriptyline (Elavil)	d-methamphetamine	MDPV	Procaine (novacaine)
Amobarbital (Amytal)	Dopamine HCl	**Meperidine (Demerol)**	Promethazine
Amyl nitrite	Doxylamine succinate	Mephedrone HCl	Propranolol HCl
Atropine	d-propoxyphene (Darvocet)	Mescaline	Proxymetacaine
Benzocaine	Duloxetine (Cymbalta)	Methadone	Pseudoephedrine
*Benzoylecgonine*	*Ecgonine methyl ester*	Methaqualone (Quaalude)	Psilocin
Benzylpiperazine	*EDDP perchlorate*	Methcathinone	Quetiapine fumarate (Seroquel)
Buprenorphine	*EMDP HCl*	Methicillin	Quinine
Bupropion	Erythromycin	Methylone HCl	Risperidone (Risperidol)
Caffeine	Estazolam	**Methylphenidate (Ritalin)**	Salicylic acid
Cannabidiol	Etomidate	Midazolam (Dormicum)	Scopolamine
*Cannabinol*	Excedrin pill	*Morphine glucuronide*	Serotonin HCl
Carbamazepine	Fenfluramine (Fen-Phen)	Morphine	Sertraline HCl (Zoloft)
Carisoprodol	Fentanyl	Naltrexone HCl	Sildenafil citrate (Viagra)
Celecoxib (Celebrex)	Flunitrazepam (Rohypnol)	Naproxen	Sodium pentothal
Chloral hydrate	Fluoxetine HCl (Prozac)	*n-desmethyltramadol HCl*	Strychnine
Chloramphenicol	Flurazepam (Dalmane)	Nicotine	Sulfadoxine
Chlordiazepoxide (Librium)	Haloperidol (Haldol)	*Norcocaine HCl*	Temazepam (Restoril)
Chlorpheniramine	**Heroin**	*Norcodeine*	Tetracaine (amethocaine)
Chlorpromazine HCl (Thorazine)	Hydrochlorothiazide	Nordiazepam	Tetracycline
Ciprofloxacin	**Hydrocodone**	Olanzapine	*Theophylline*
cis-Tramadol HCl	Hydromorphone (Dilaudid)	Oxazepam	Trazodone
Citalopram HBr	Ibogaine	*Oxazepam glucuronide*	Triazolam (Halcion)
Clonazepam (Klonopin)	**Ibuprofen**	**Oxycodone (Oxycotin)**	Vancomycin
Clonazipine	Inositol	Oxymorphone	Zaleplon (Sonata)
*Cocaethylene*	Isoniazide	*Paraxanthine*	Zolpidem tartrate (Ambien)
Cocaine base	Ketamine	**PCP**	Zopiclone (Lunesta stereoisomer)

**Table 2.** Spectral search and match results for drugs in saliva (except oxycodone in water).

**Unknown**	**Oxycodone (0.1 mg/mL water)**		**Cocaine (50 ng/mL)**		**PCP (1 mcg/mL)**		**Diazepam (1 mcg/mL)**		**Acetaminophen (10 mcg/mL)**	
**Rank**	**HQI**	**Chemical**	**HQI**	**Chemical**	**HQI**	**Chemical**	**HQI**	**Chemical**	**HQI**	**Chemical**
1	0.073	Oxycodone	0.287	Cocaine	0.019	PCP	0.022	Diazepam	0.276	Acetaminophen
2	0.734	Hydrocodone	0.348	Ethyl-benzoyl-ecgonine	0.315	Fentanyl	0.317	Tema-zepam	0.639	Sulfadoxine
3	0.800	Trazadone	0.349	Benzoyl-ecgonine	0.325	EMDP*	0.328	Nor-diazepam	0.704	Serotonin

* EMDP is 2-Ethyl-5-methyl-3,3-diphenylpyrroline.
